# Supplementary material for: Short-term interaction between silent and devastating earthquakes in Mexico
Source: Nat Commun. 2021 Apr 12;12:2171. doi: 10.1038/s41467-021-22326-6 (PMC8042113; doi:10.1038/s41467-021-22326-6)
Supplement: Supplementary file 1 — Supplementary Information [file 41467_2021_22326_MOESM1_ESM.pdf]

nature COMMUNICATIONS

Supplementary Figures

Short-Term Interaction between Silent and Devastating Earthquakes in Mexico

Authors: V. M. Cruz-Atienza*, J. Tago, C. Villafuerte, M. Wei,

R. Garza-Girón, L. A. Dominguez, V. Kostoglodov, T. Nishimura,

S. I. Franco, J. Real, M. A. Santoyo, Y. Ito and E. Kazachkina.

*Correspondence to: cruz@geofisica.unam.mx

This PDF file includes:

Supplementary Fig. 1. GPS displacement data.

Supplementary Fig. 2. Aseismic-slip time evolution for different depth ranges.

Supplementary Fig. 3. Aseismic slip and CFS preceding the Mw7.1 Puebla-Morelos earthquake.

Supplementary Fig. 4. Plate interface aseismic slip and CFS preceding the Mw7.2 Pinotepa earthquake.

Supplementary Fig. 5. Template-matching seismic detections preceding the Mw7.2 Pinotepa earthquake.

Supplementary Fig. 6. Template-matching earthquake magnitude determination and seismicity analysis.

Supplementary Fig. 7. Peak amplitudes of the Mw8.2 surface waves as a function of epicentral distance.

Supplementary Fig. 8. Validation of the plate-interface dynamic perturbation estimates.

Supplementary Fig. 9. Plate-interface dynamic perturbations for the Mw8.2 Tehuantepec earthquake.

Supplementary Fig. 10. Discontinuous Galerkin 3D earthquake simulations.

Supplementary Fig. 11. Rate-and-state friction model for Oaxaca SSEs.

A

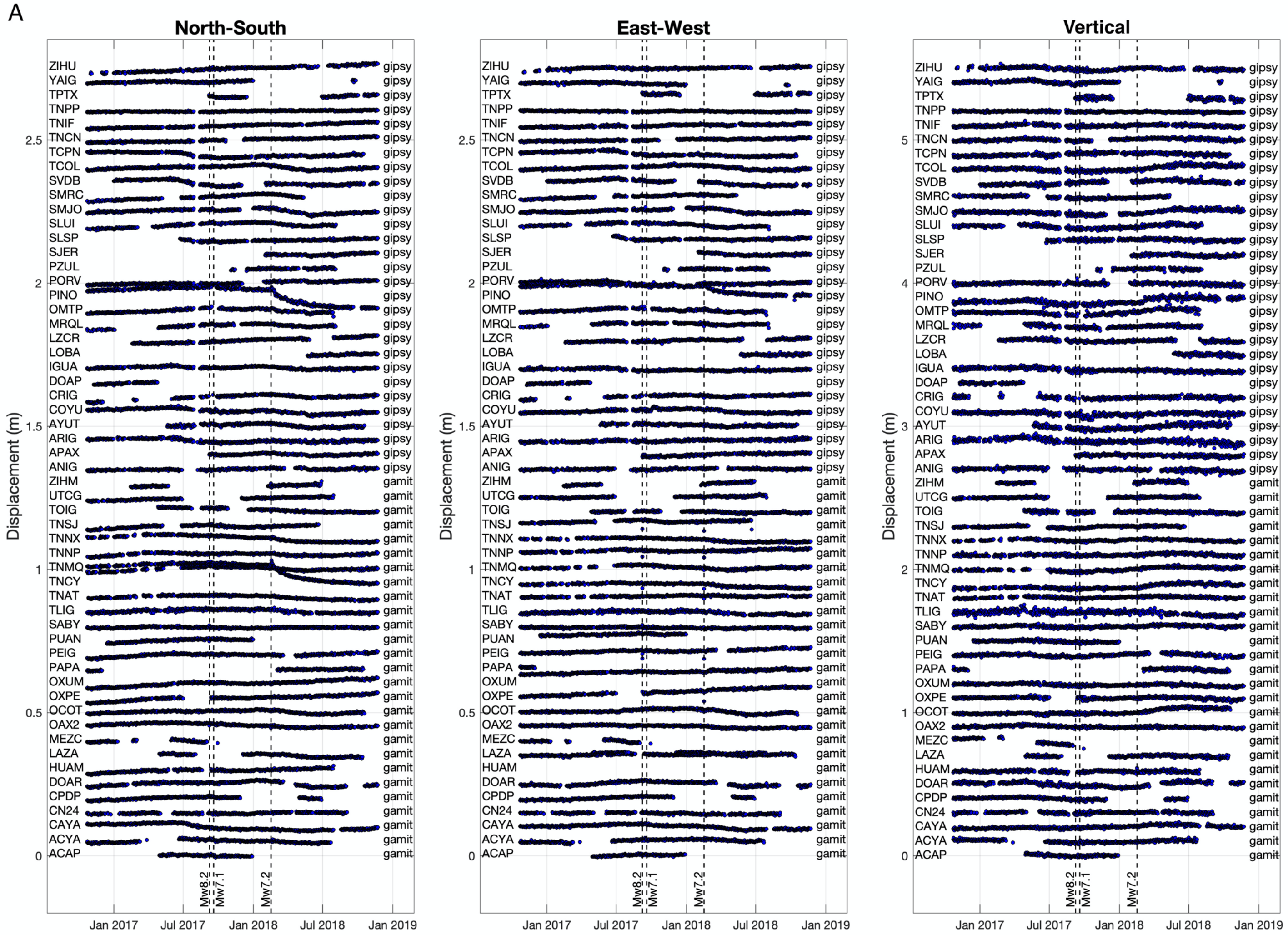

Supplementary Fig. 1. Displacement GPS time series used in the study. 57 selected stations (A) from October 23 (2016) to November 22 (2018) and (B) from November 22 (2018) to October 8 (2019). To the right of each series is indicated the data processing method selected for the inversions. Vertical dashed lines indicate the occurrence of the three earthquakes of the sequence.

nature COMMUNICATIONS

B

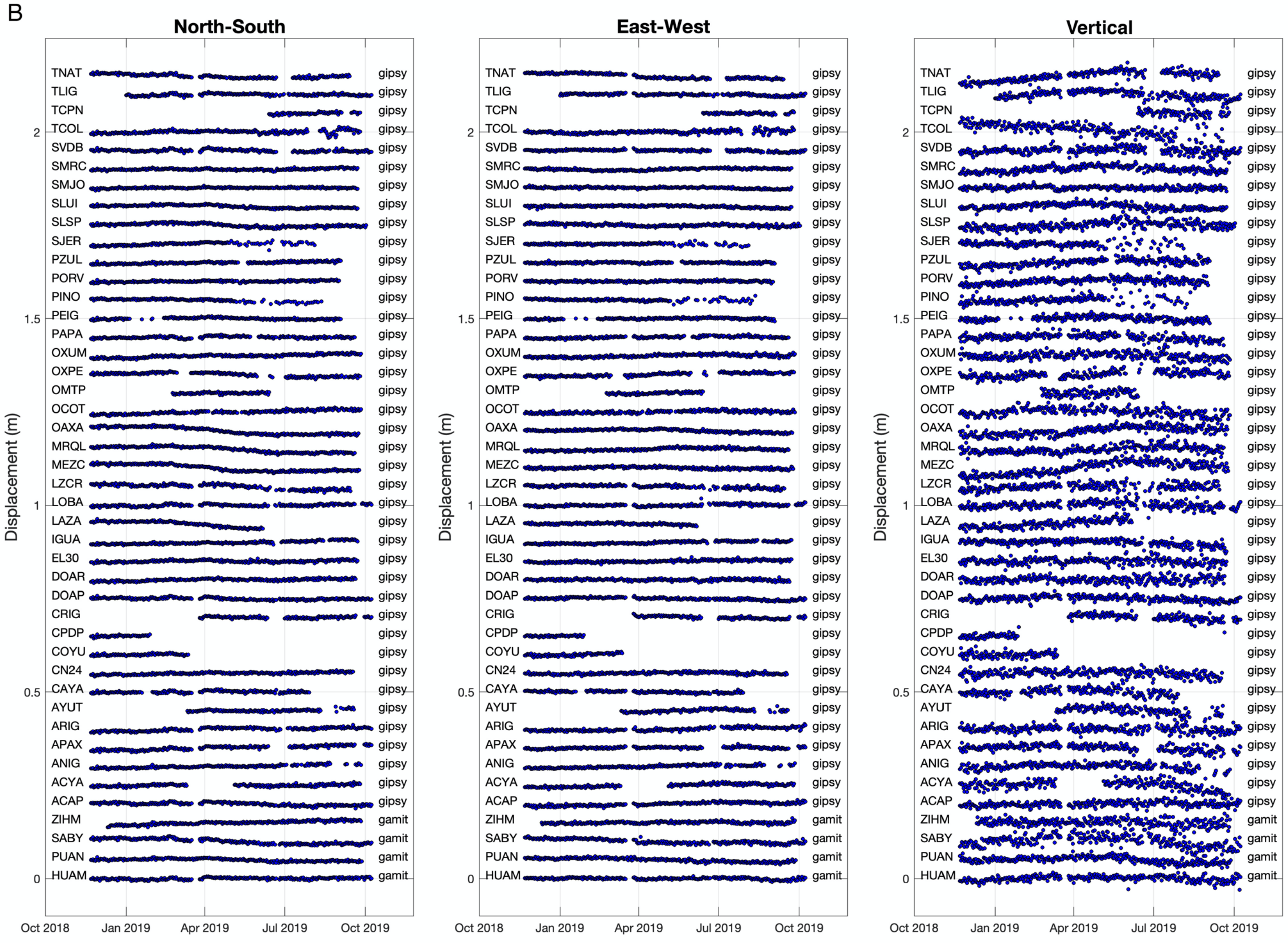

Supplementary Fig. 1. (Continuation).

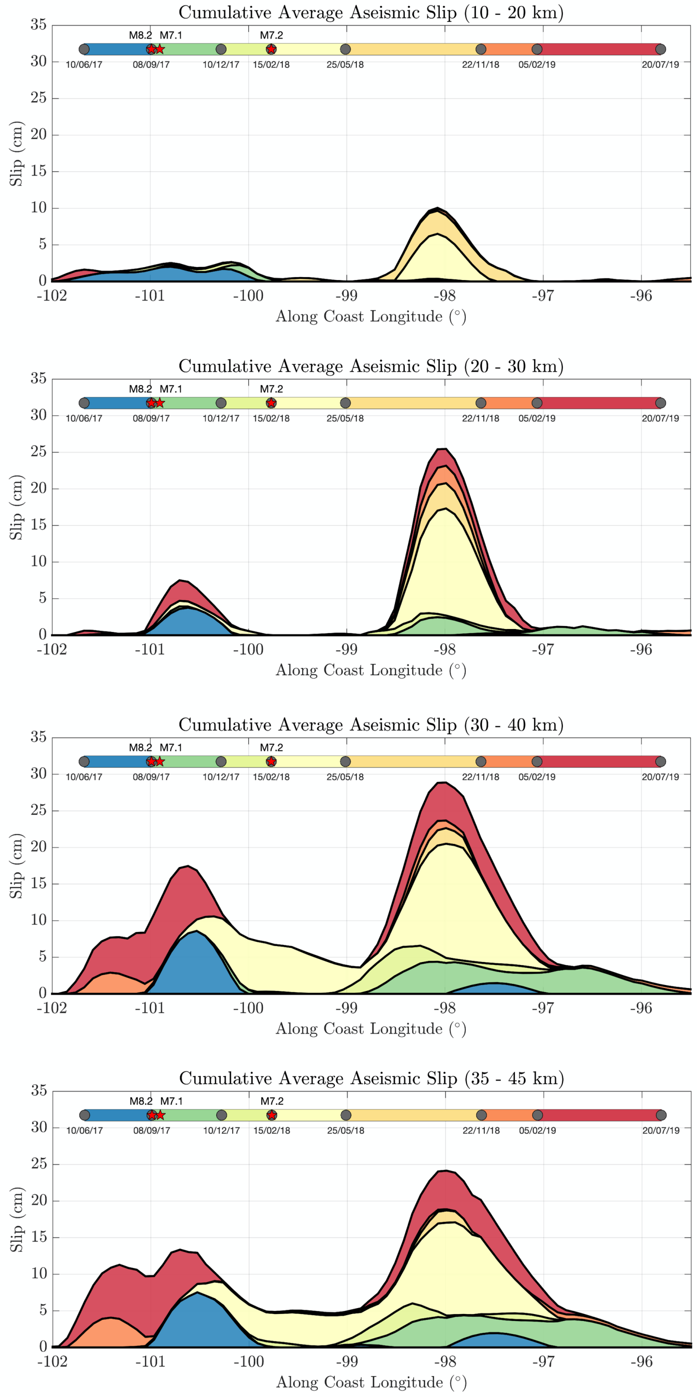

Supplementary Fig. 2. Cumulative aseismic slip evolution over different plate-interface depth ranges (see panel titles). Average values per depth range were obtained from solutions shown in Figure 3A. Between 10 and 20 km depth (i.e., mainly offshore), only the G-SSE1 and the PE-afterslip are significant, with maximum slip of 2.0 and 6.5 cm, respectively. The largest SSE activity concentrates between 20 and 45 km depth.

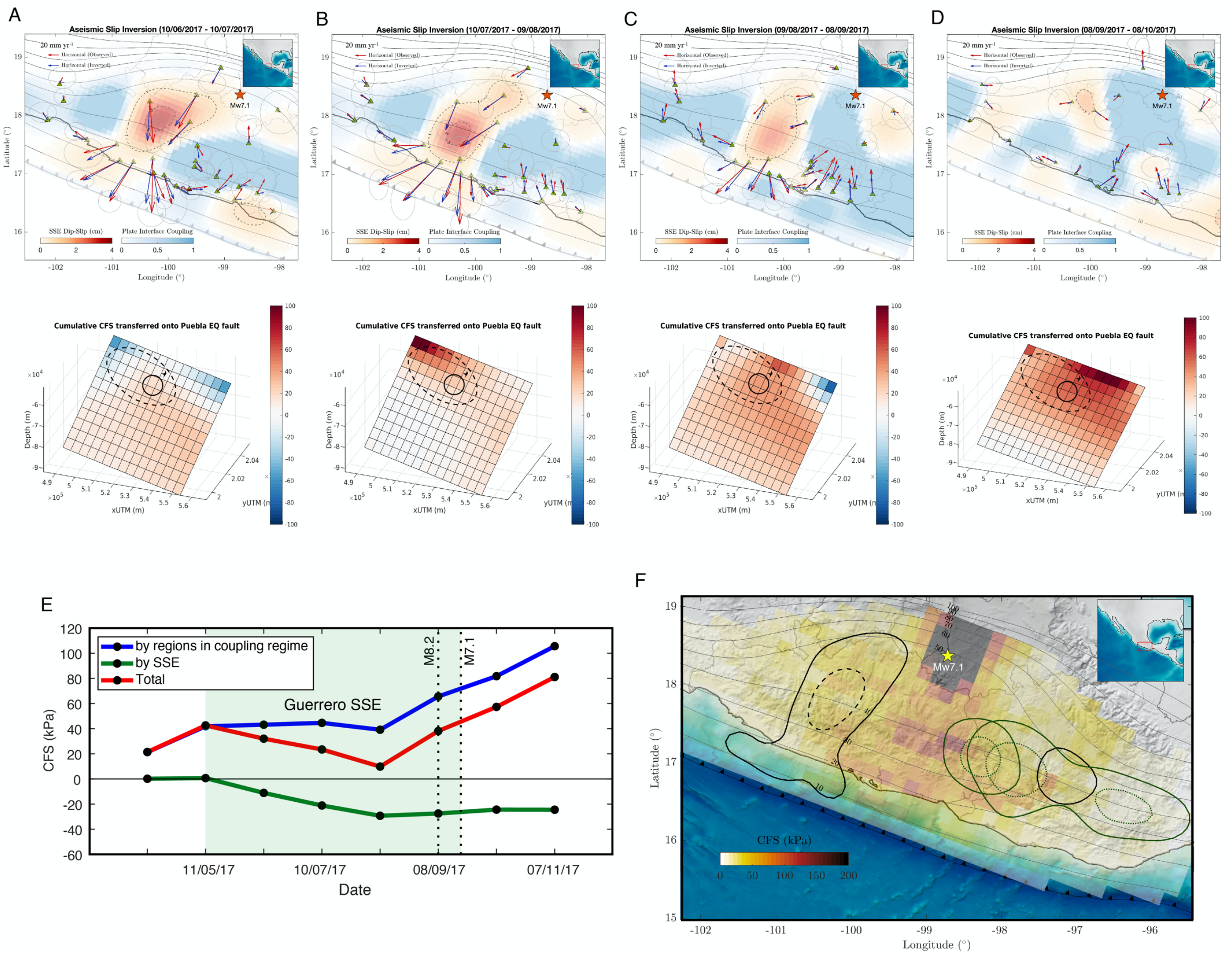

Supplementary Fig. 3. Interaction between the GSSE-1 and the Puebla-Morelos earthquake. (A-D) 30-day time windows aseismic slip inversions of the G-SSE1 (up) and the associated cumulative total CFS over the intermediate-depth normal fault where the Mw7.1 Puebla-Morelos earthquake took place on September 19, 2017 (down). The inverted time windows are shorter than those shown in Figure 2. (E) CFS evolution within a 20 km radius from the Puebla-Morelos hypocenter. Notice the CFS sustained growth induced by the PIC in the later SSE stage. (F) Dynamic CFS maximum values on the plate interface induced by the Puebla-Morelos earthquake seismic waves. They were estimated with a 3D finite source simulation (see Supplementary Fig. 10) similar to that performed for the Pinotepa earthquake (Fig. 6C) but using the finite-source solution determined by Mirwald et al. (*1*). Aseismic slip events right before the earthquake are shown with black contours, while those occurred immediately after the earthquake are shown with green contours.

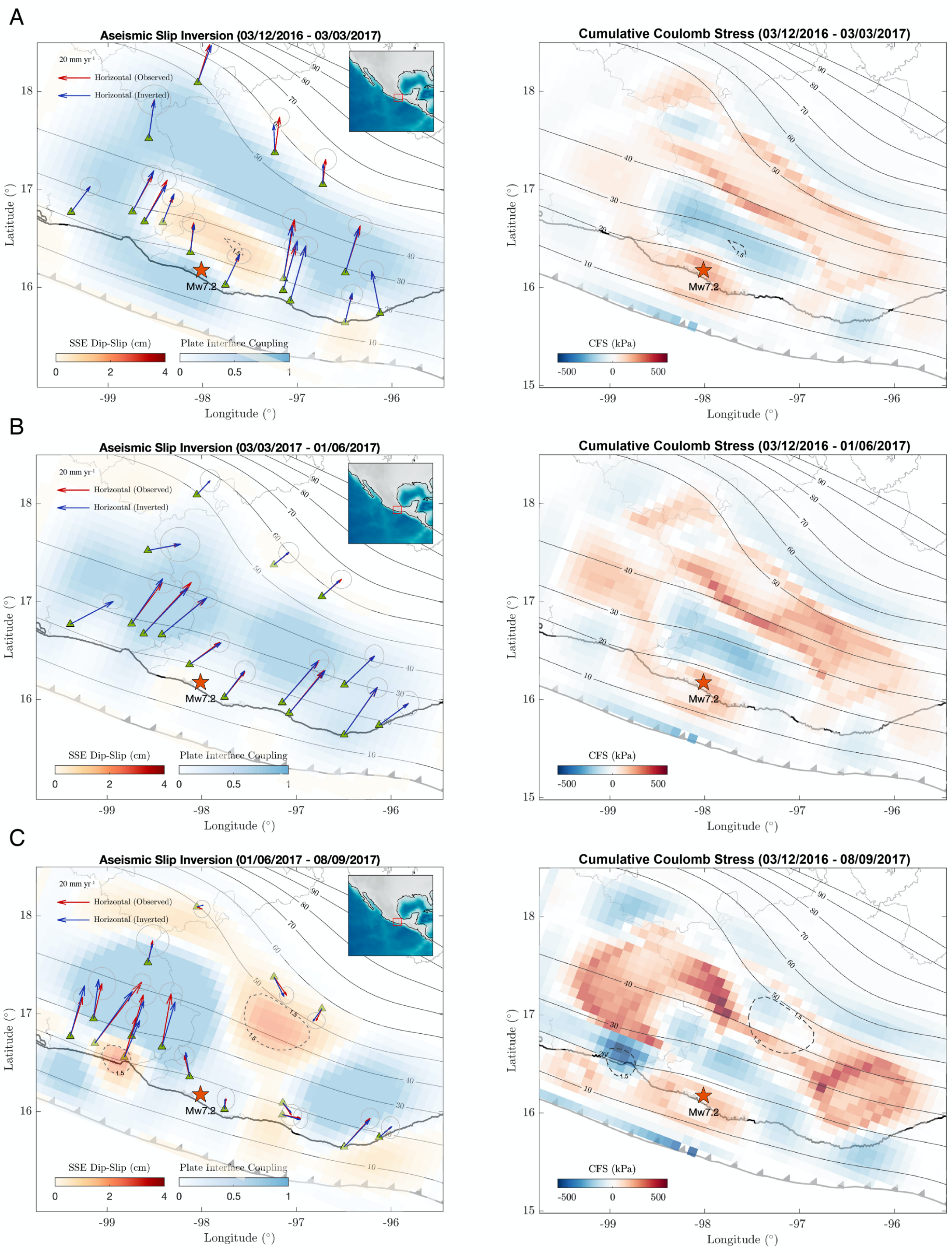

Supplementary Fig. 4. Aseismic slip inversions preceding the Mw7.2 Pinotepa earthquake during the O-SSE1 (left column) and the associated cumulative CFS on the plate interface (right column). Dashed contours in the right column show the aseismic slip contours of the associated time window. Notice that the inverted time windows are shorter than those shown in Fig. 2. Cumulative CFSs from these higher time-resolution inversions are shown in Fig. 5a.

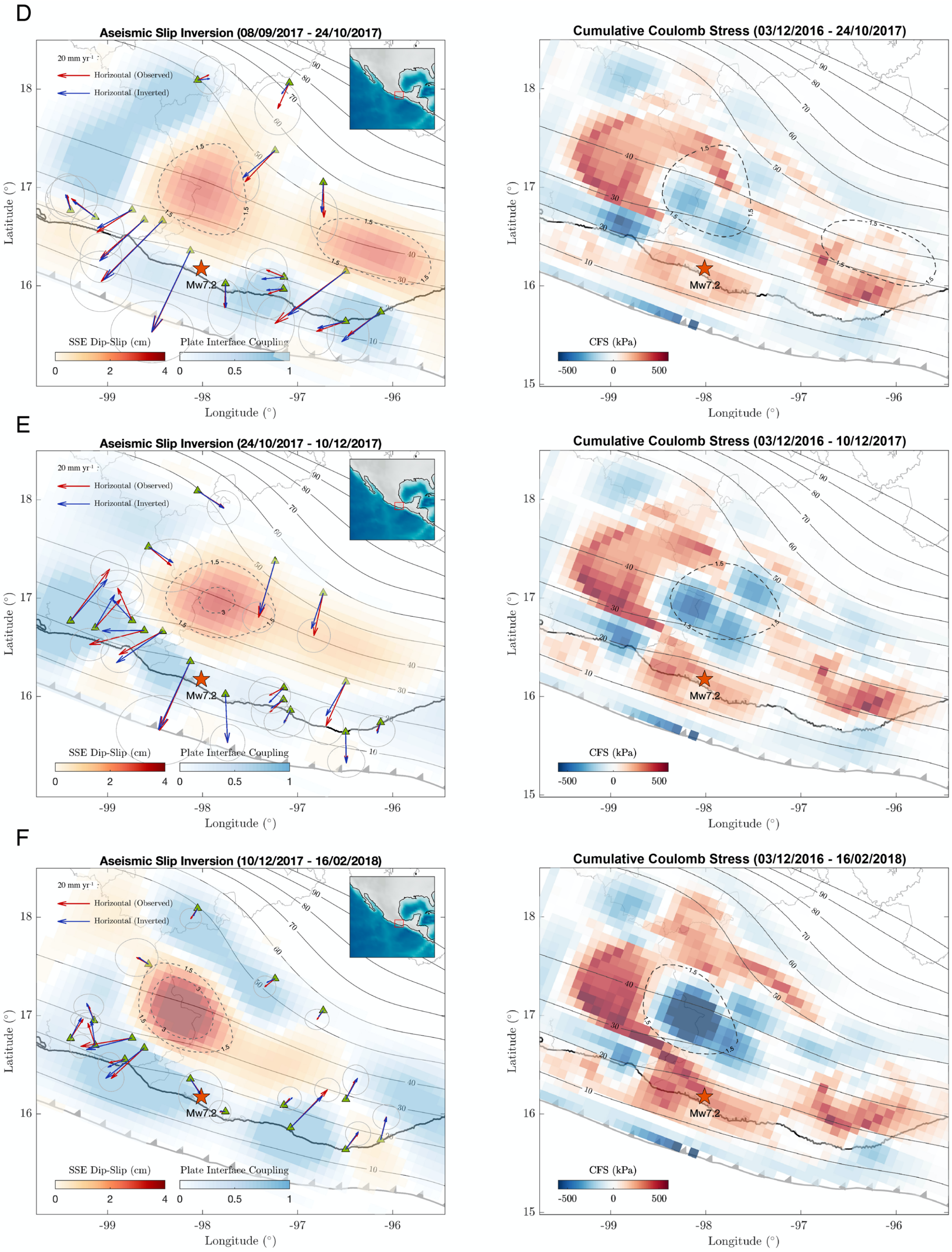

Supplementary Fig. 4. (Continuation).

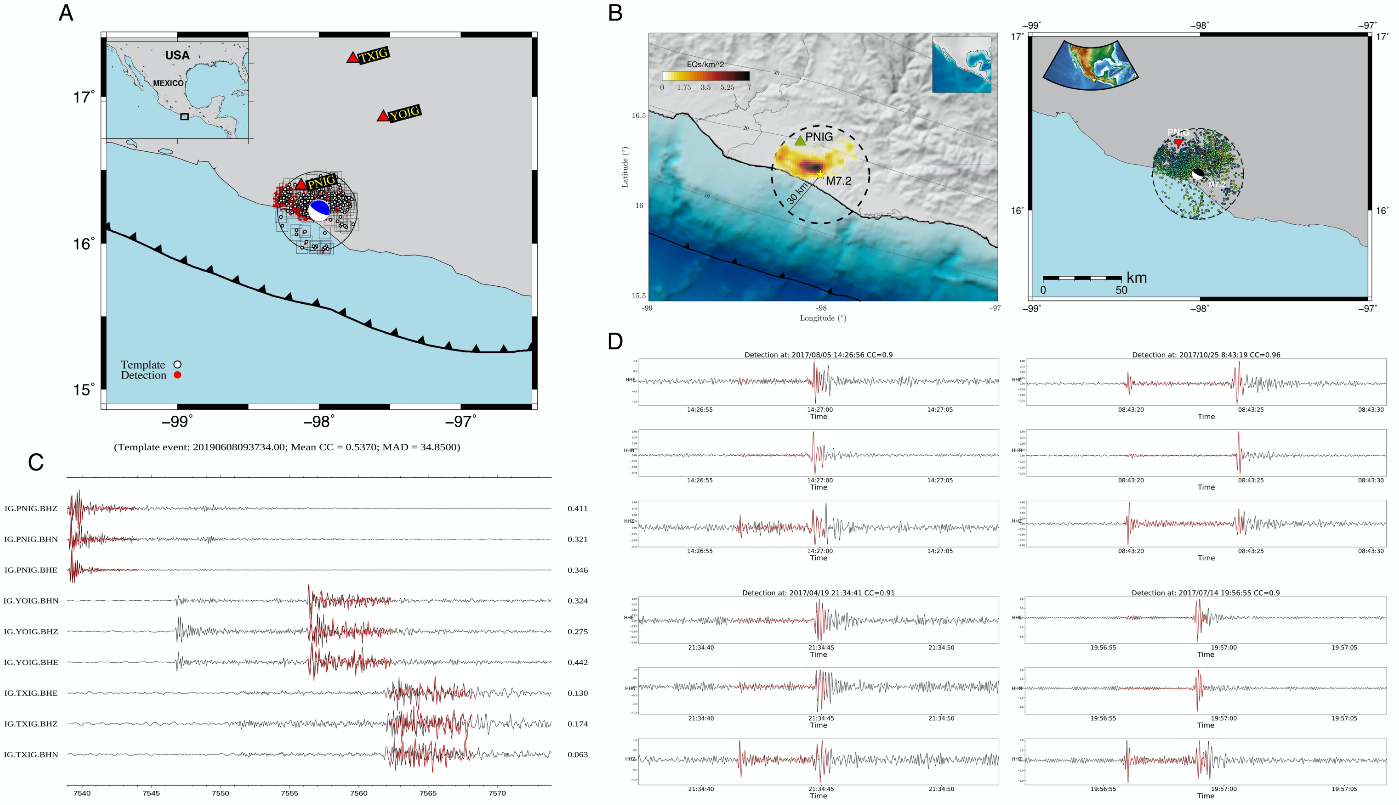

Supplementary Fig. 5. Template matching results using two different methods during the year preceding the Pinotepa earthquake. (A) Map of events detected by method 1 using three stations at a regional scale. (B) Density map for the template events used by method 2 (left) and their spatial distribution (right). (C) Example of a regional detection made at stations PNIG, YOIG and TXIG using method 1 for the direct S wave and its coda. (D) Examples of local detections made at station PNIG using method 2. Fits of the templates with both the P and S direct waves along with the coda of the P waves guarantee that detections come from the same hypocentral locations as the template events. See Figure 5c of the main text.

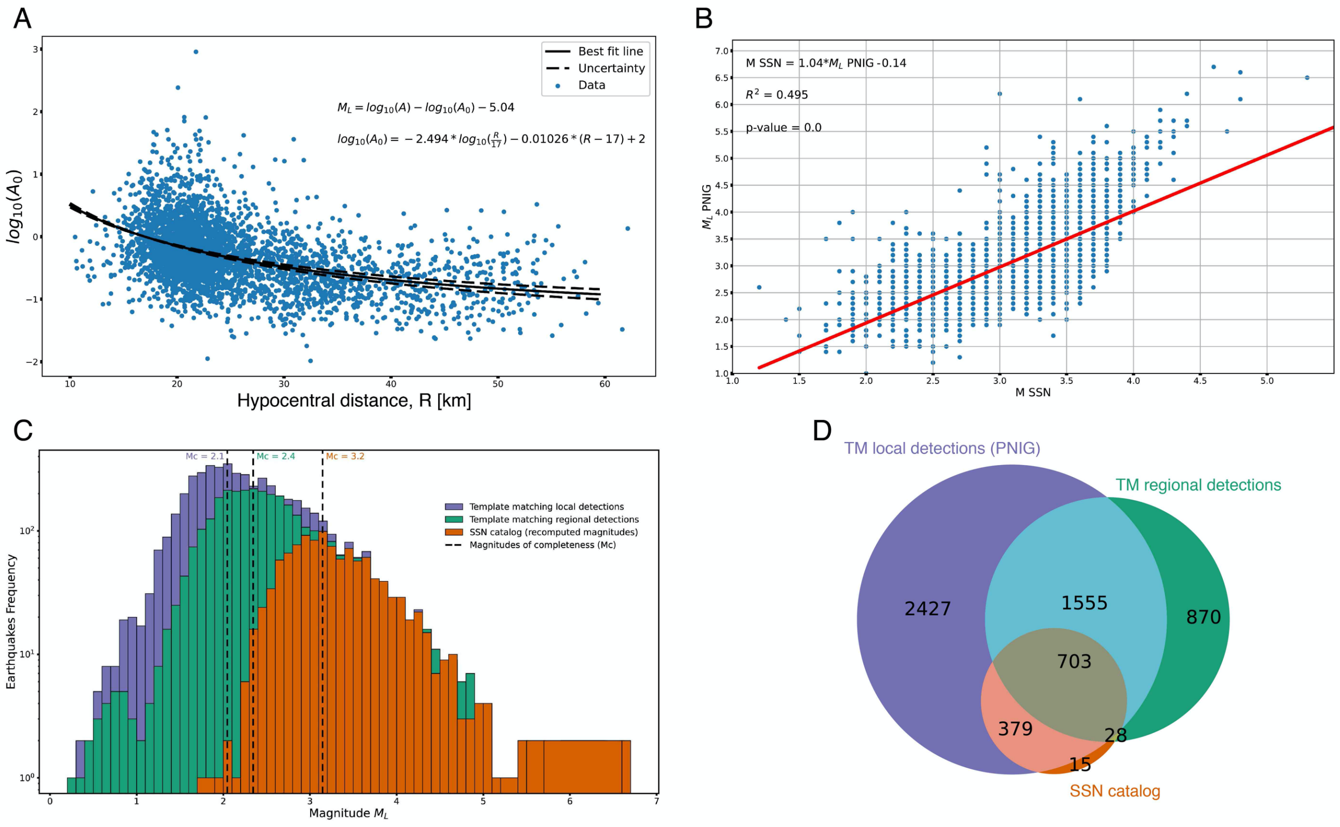

Supplementary Fig. 6. Magnitude estimation for the template-matching detected earthquakes and final catalogs comparison. (A) Attenuation relationship calculated on the horizontal components (geometric mean) of PNIG and magnitude scale M_L. (B) Correlation between recomputed M_L magnitudes using the PNIG station and the magnitudes reported by the SSN. (C) Earthquake frequency distributions for the template matched catalogs using the closest station PNIG (blue), three stations of the regional network (green) and the catalog provided by the SSN (orange). (D) Venn diagram showing the relationship of the number of events of each catalog. The intersections are calculated by finding common events in time (events within 10 seconds of each other).

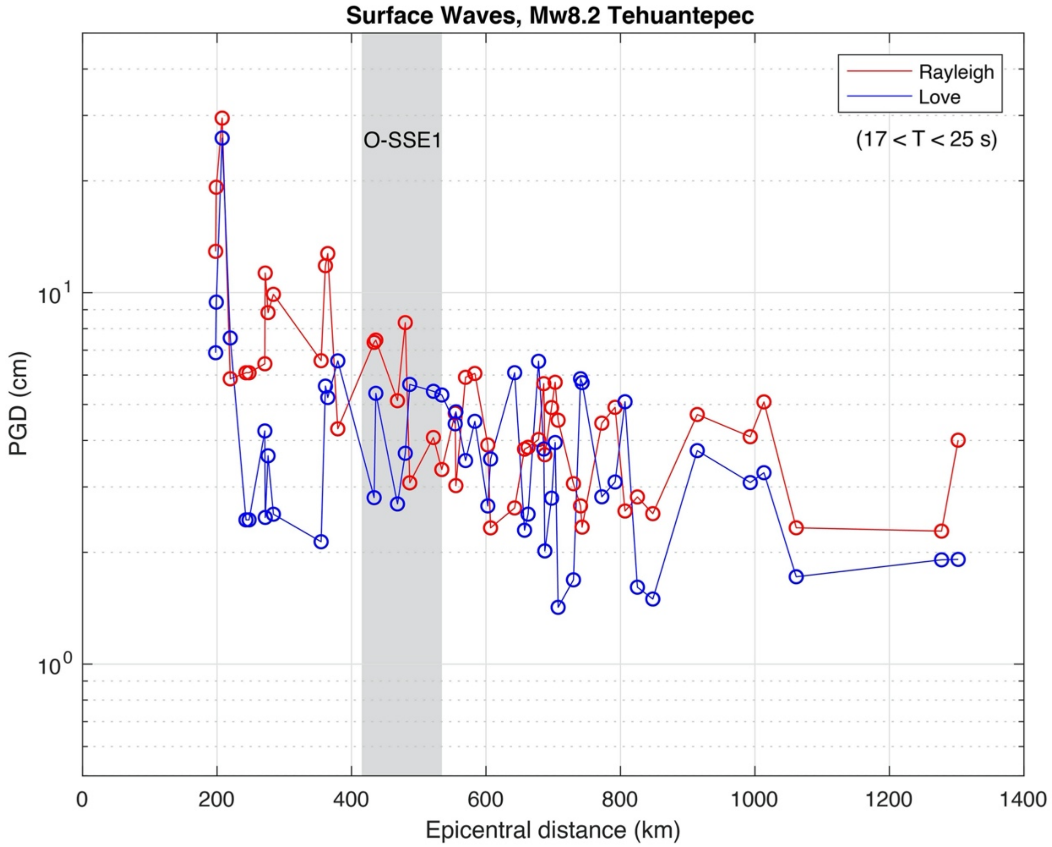

Supplementary Fig. 7. Attenuation of peak ground displacements for Rayleigh and Love waves produced by the Mw8.2 Tehuantepec earthquake. Peak values of both types of surface waves differ in less than a factor of two at distances where the O-SSE1 was developing at the moment of the earthquake.

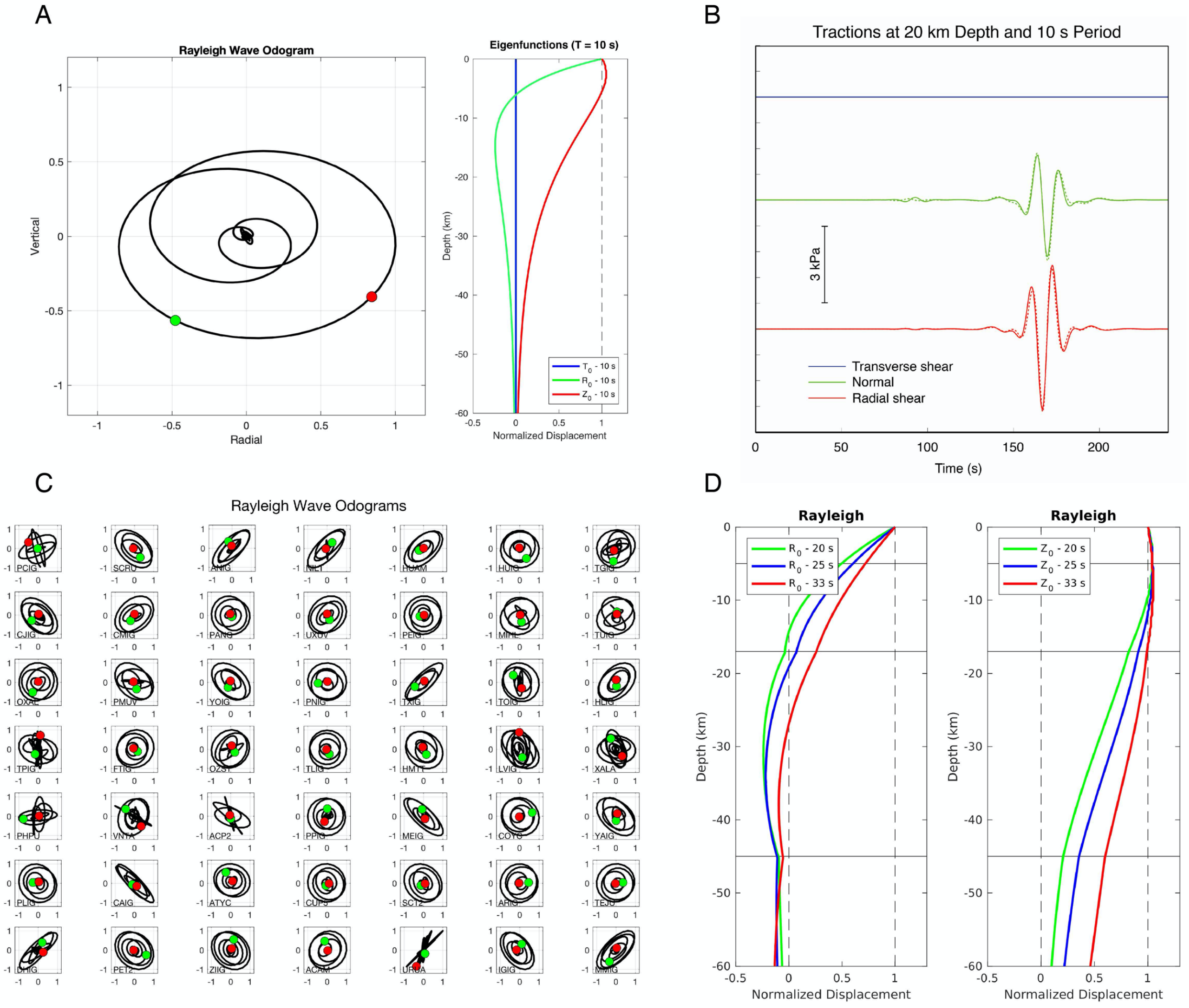

Supplementary Fig. 8. Validation of the procedure to estimate dynamic perturbations on the plate interface from actual strong motion records. (A) Odogram of a Lamb pulse (i.e., of the wavefield produced by a vertical force applied at the free surface of a homogeneous halfspace) (left), and corresponding eigenfunctions for the Rayleigh wave fundamental mode at 10 s period (right). (B) Comparison at 20 km depth (horizontal plane) and 10 s period of the exact traction evolution (solid) and the estimated traction following the procedure described in Methods (dashed). (C) Odograms for the radial and vertical displacement components around 25 s period from actual records of the Mw8.2 Tehuantepec earthquake on 49 strong motion station (see Supplementary Fig. 9). (D) Eigenfunctions of the Rayleigh waves fundamental mode in a crustal 1D velocity model (*2*) used to estimate beneath each station the traction, CFS and dilation evolution on the 3D plate interface shown in Fig. 6A and Supplementary Fig. 9.

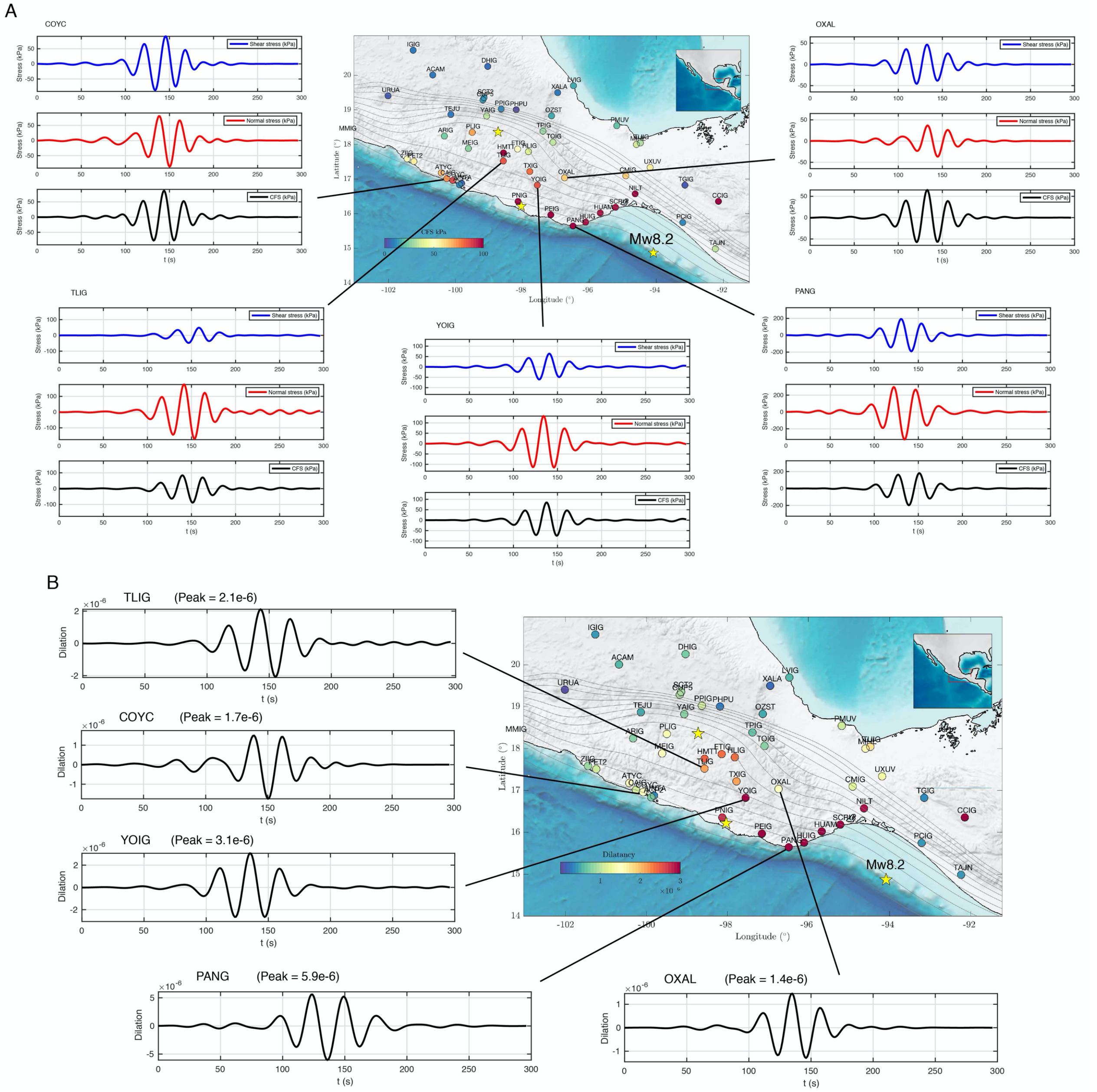

Supplementary Fig. 9. Dynamic perturbations at the plate interface produced by the Mw8.2 Tehuantepec earthquake. 25 s period dynamic-stress (A) and dilation (B) perturbations over the 3D plate interface (gray contours) estimated from actual strong motion records of the earthquake below different seismic stations (circles). CFSs (computed in the plate-convergence slip direction) and dilations peak values are color-coded in each site. Values where there is no plate interface below correspond to 50 km depth over a horizontal surface.

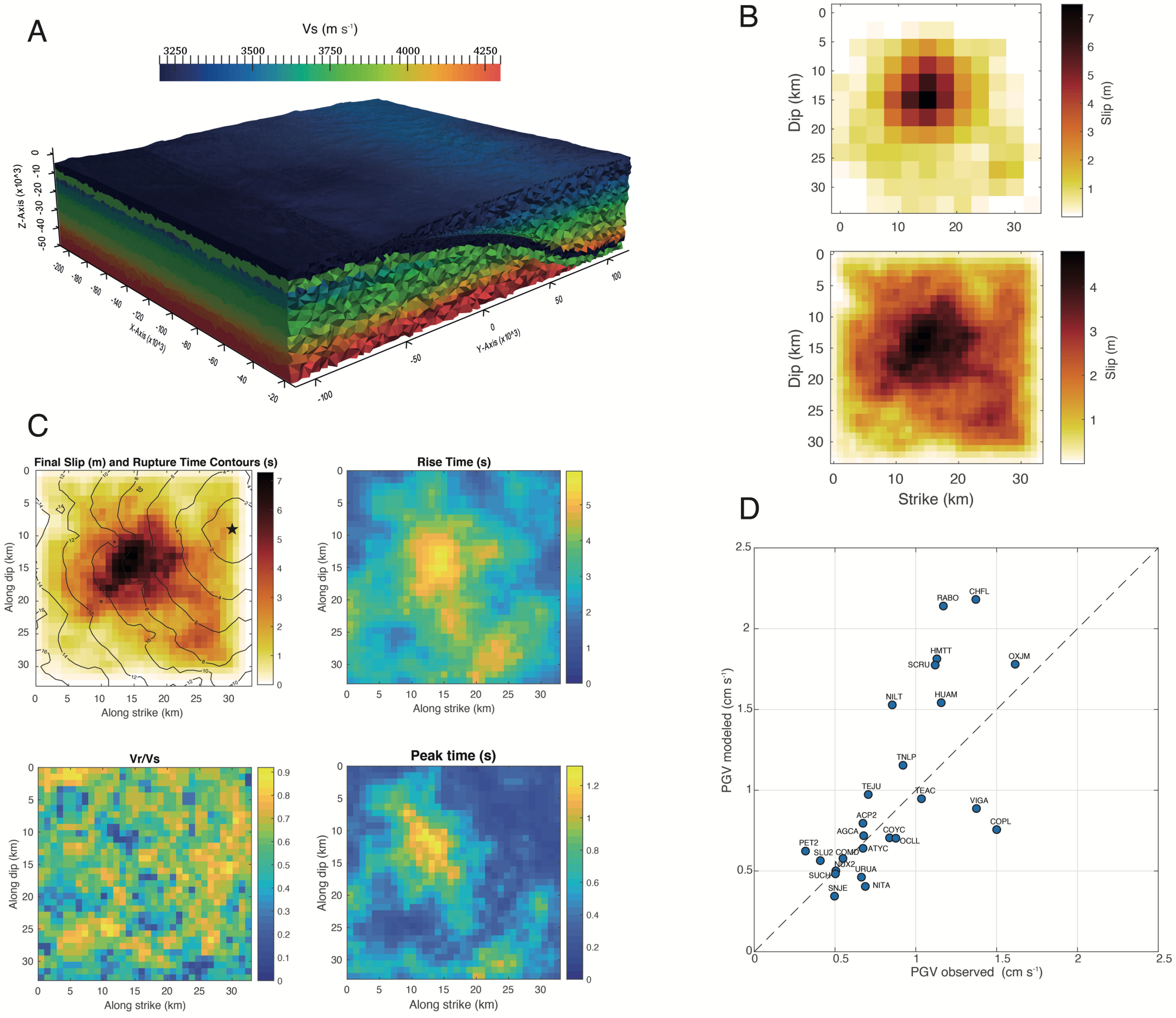

Supplementary Fig. 10. 3D kinematic finite-source numerical simulation of the Mw7.2 Pinotepa earthquake. (A) Non-structured tetrahedral discretization of the Oaxaca subduction zone. (B) Initial USGS finite source inversion (up) and broad-band wavelength finite slip model (bottom). (C) Spatial distribution of the slip, the rise time, the rupture velocity and the peak time used to describe the kinematic rupture evolution. (D) Comparison between modeled and observed horizontal PGV for different hard-site strong motion stations (see Supplementary Fig. 9 for site locations).

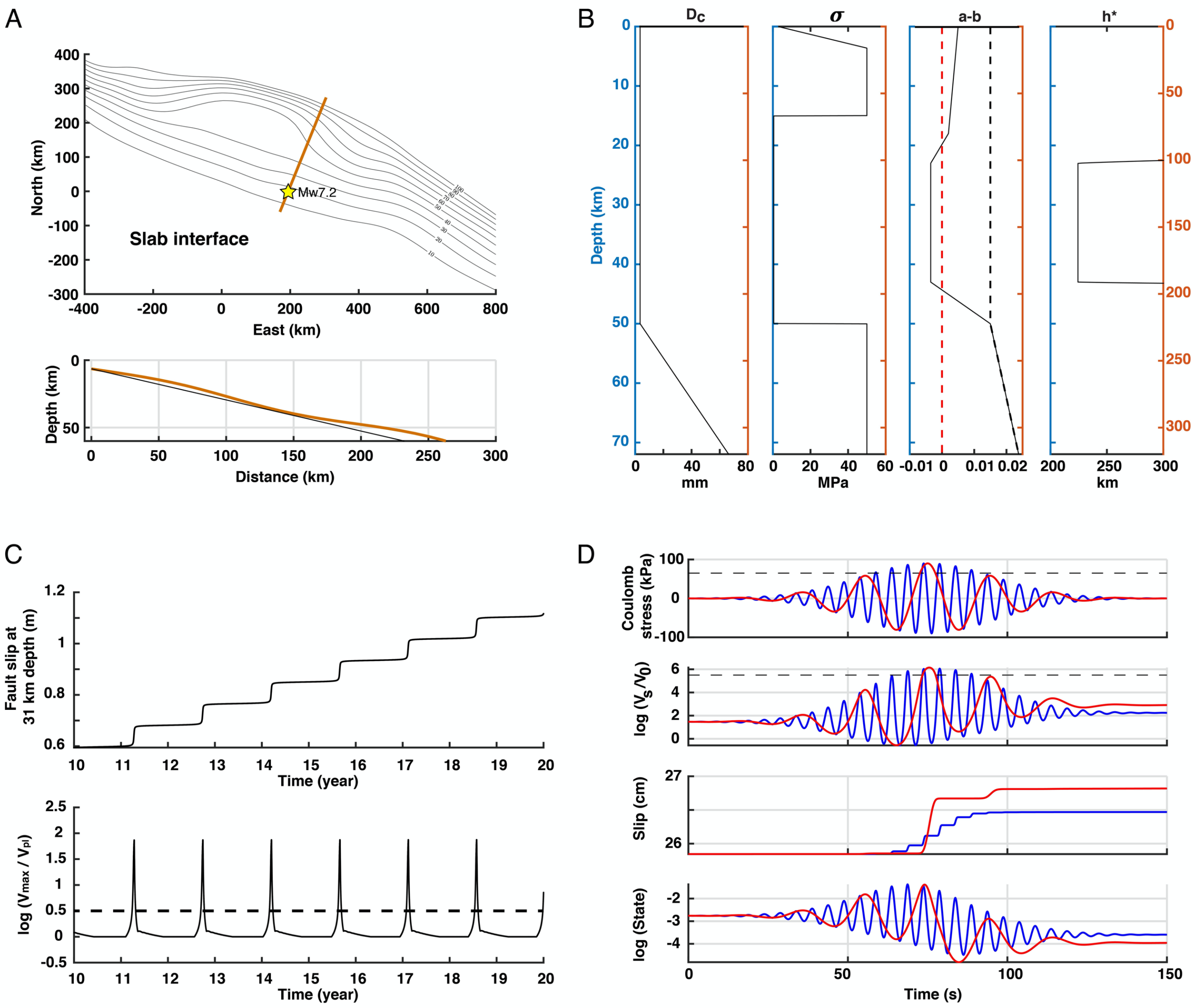

Supplementary Fig. 11. Rate-and-state friction SSE model for the Oaxaca state. (A) Top, slab geometry in the study area across the 2018 Mw7.2 Pinotepa earthquake epicenter. The orange solid line shows the profile of our 2D model over the 3D plate interface geometry. Bottom, the black solid line shows the planar fault model and the orange line shows the slab geometry. (B) Rate-and-state parameters used in our 2D reference model. Dc above 50 km is 3.5 mm. The effective normal stress in the low stress zone is 0.45 MPa. (C) Slip at 31 km depth and the maximum slip rate on the fault for the reference model. (D) Evolution of key model parameters with two different perturbations with different characteristic periods (5 s and 20 s periods) and same 60 kPa CFS peak values. Notice

that longer period waves have significantly larger SSE triggering potential, i.e., ~40% larger final slip and much larger (effective) slip acceleration.

Supplementary References

1. A. Mirwald *et al.*, The 19 September 2017 (Mw7.1) Intermediate-Depth Mexican Earthquake: A Slow and Energetically Inefficient Deadly Shock. *Geophys Res Lett* **46**, 2054-2064 (2019).
2. M. Campillo, S. K. Singh, N. Shapiro, J. Pacheco, R. B. Hermann, Crustal structure south of the Mexican volcanic belt, base on group velocity dispersion. *Geofis Int* **35**, 361-370 (1996).
