# Supplementary material for: Short-term interaction between silent and devastating earthquakes in Mexico
Source: Nat Commun. 2021 Apr 12;12:2171. doi: 10.1038/s41467-021-22326-6 (PMC8042113; doi:10.1038/s41467-021-22326-6)
Supplement: Supplementary file 3 — Description of Additional Supplementary Files [file 41467_2021_22326_MOESM3_ESM.pdf]

Description of Additional Supplementary Files

Supplementary Movie 1

Evolution of aseismic slip rate (i.e., SSEs and PIC) at the plate interface across the states of Guerrero and Oaxaca, Mexico. Dashed line areas depict large historical thrust earthquakes.
